# Rare coding variants from ADSP R5 whole-genome sequencing implicate novel genes in Alzheimer’s disease

**DOI:** 10.21203/rs.3.rs-9013646/v1

**Published:** 2026-03-31

**Authors:** Wan-Ping Lee, Hui Wang, Yuk Yee Leung, Po-Liang Cheng, Wendi Zheng, Otto Valladares, Wei-Hsuan Chung, Amanda Kuzma, Adam Naj, Badri Vardarajan, Anthony Grsiwold, Jonathan Haines, Li-San Wang, Gerard Schellenberg

**Affiliations:** University of Pennsylvania; University of Pennsylvania; Taichung Veterans General Hospital; Columbia University; University of Pennsylvania; University of Pennsylvania; Penn Neurodegeneration Genomics Center, Department of Pathology and Laboratory Medicine, University of Pennsylvania Perelman School of Medicine, Philadelphia, Pennsylvania; University of Pennsylvania; Department of Neurology, College of Physicians and Surgeons, Columbia University and the New York Presbyterian Hospital; The John P. Hussman Institute for Human Genomics, University of Miami; Case Western Reserve University; University of Pennsylvania; University of Pennsylvania

## Abstract

The Alzheimer’s Disease Sequencing Project (ADSP) Release 5 provides whole-genome sequencing data from 58,507 individuals across diverse ancestries to discover rare coding variants and genes associated with Alzheimer’s disease (AD) and AD-related traits. Gene-based aggregation tests identified 40 genes surpassing a Bonferroni-corrected gene-wide significance threshold, including established loci (TREM2, PSEN1) and putative novel candidates. In replication analyses, 21 genes showed nominal support in UK Biobank and Alzheimer’s Disease Genetics Consortium (ADGC) cohorts, with eight genes (TREM2, ACADS, MFSD12, NUP210L, PIEZO2, PSEN1, SMURF2, AKAP13) supported under identical masks. Carrier-based analyses of AD-related traits linked rare variants to age at onset, neuropathology, cognition, and cerebrospinal fluid biomarkers (Aβ42, total tau, pTau181). Furthermore, we observed that AD-enriched variants were more likely to be ancestry-concentrated, and coalescent analyses indicated that AD risk alleles are younger than background variants. Together, these findings provide a multi-ancestry rare-variant resource for AD gene discovery.

## Introduction

Alzheimer’s disease (AD) is the leading cause of dementia, affecting more than 6 million individuals in the United States and over 50 million worldwide^1^. With aging populations, prevalence and associated societal costs are expected to rise substantially in the coming decades. Genome-wide association studies have identified many common-variant loci for AD, yet much of the genetic architecture remains unresolved, particularly for rare variation and alleles with larger effects that are incompletely captured by array-based approaches and imputation. Large-scale sequencing studies are therefore essential to identify rare risk and protective variants, implicate effector genes and pathways, and refine biological mechanisms that can inform therapeutic strategies.

The Alzheimer’s Disease Sequencing Project (ADSP), supported by the National Institute on Aging at the National Institutes of Health, was established to accelerate discovery of AD genetic determinants through whole-genome (WGS) and whole-exome (WES) sequencing across multiple cohorts. The ADSP has released successive high-quality callsets from WGS (R1, R3, and R4)^2–4^ and WES (R2)^5^, each expanding sample size, improving variant quality control, and increasing ancestral diversity. The most recent release, R5, is the largest ADSP resource to date, comprising nearly half a billion high-confidence variants from 58,507 sequenced individuals spanning multiple ancestries, and represents one of the largest single-disease sequencing callsets available for AD genetic discovery.

A central advantage of R5 is the ability to interrogate rare variation not only for case-control AD risk but also across a broad set of AD-related quantitative and neuropathological traits, including age at onset, cognitive measures, cerebrospinal fluid biomarkers (Aβ_42_, total Tau [t-Tau], and phosphorylated tau 181 [pTau_181_]), and detailed neuropathologic measures (Braak stage, CERAD neuritic plaque score, hippocampal sclerosis, TDP-43 pathology, and features of cerebrovascular disease). Joint analysis across these correlated traits can increase power to detect gene-level associations and clarify the biological processes that link genetic variation to disease manifestations. Here, we conduct gene-based rare-variant association analyses across AD diagnosis and multiple AD-related traits in ADSP R5, providing a resource for the community and yielding insights into the contribution of rare coding variation to AD pathogenesis.

## Results

### Samples, Variants, and Phenotype

The ADSP R5 dataset includes 58,507 whole-genome sequences from 57 cohorts (**Table S1**). After removing individuals related at the second degree or closer, 50,489 unrelated samples remained. Starting from the preview callset (NIAGADS ng00067.v16), and after applying quality control, 449,265,450 variants were retained in these samples. Across the callset, 51.39% of variants are singletons (allele count = 1), and 97.42% have an allele frequency < 0.01.

AD case-control diagnosis was available for 50,489 unrelated ADSP R5 participants. Of these, 12,078 were diagnosed with AD (23.9%), and 26,308 were controls (52.1%). We excluded individuals with mild cognitive impairment, family-reported AD, possible or probable AD, other dementias, and progressive supranuclear palsy from association analyses. AD case and control counts by genetic ancestry (inferred using GrafAnc^6^) were: African (89/679), African American (1,338/2,842), East Asian (921/1,335), South Asian (12/2,445), European (6,757/11,537), Latin American 1 (2,053/3,853), Latin American 2 (676/2,749), Middle Eastern and North African (34/30), Native American (149/693), and multi-ancestry (49/145).

In addition to AD case-control status, we analyzed AD-related quantitative and neuropathologic traits (hereafter referred to as AD-related traits/endophenotypes) harmonized by the ADSP Phenotype Harmonization Consortium^7^, including age at onset, Braak stage, cognitive domain scores for memory (MEM), executive function (EXF), language (LAN), and visuospatial ability (VSP), cerebrospinal fluid biomarkers (Aβ_42_, total tau, and pTau_181_), and neuropathologic measures, including CERAD neuritic plaque score, hippocampal sclerosis (HS_S), Lewy body pathology stage (LEWY_full), TDP-43 pathology, and cerebrovascular disease traits (CVD_S, atherosclerosis [CVD_ATH], and arteriolosclerosis [CVD_ART]). Each trait was available for at least 1,000 individuals (**Table S2**). Collectively, this multi-ancestry sample and the breadth of harmonized traits enable rare-variant association testing for AD diagnosis and broad AD-relevant clinical, biomarker, cognitive, and neuropathologic traits.

### Rare-Variant Aggregation Associations Across AD and ADRelated Traits

#### Rare-Variant Aggregation Association

Using REGENIE^8^, we applied multiple rare-variant (minor allele frequency < 1%) association tests and annotation masks to improve robustness to different underlying genetic architectures, including scenarios in which most variants have effects in the same direction (burden), heterogeneous directions or magnitudes of effect (SKAT/SKAT-O)^9^, or sparse strong effects (ACAT-O/ACAT-V)^10^. We performed these tests on three VEP-based masks: M1 (stop_lost,start_lost, stop_gained, frameshift_variant, transcript_ablation), M2 (inframe insertion/deletion, missense, protein altering variants), and M3 (splice_acceptor_variant, splice_donor_variant, splice_donor_5th_base_variant, splice_region_variant, splice_donor_region_variant, splice_polypyrimidine_tract_variant).

**Figure 1:**
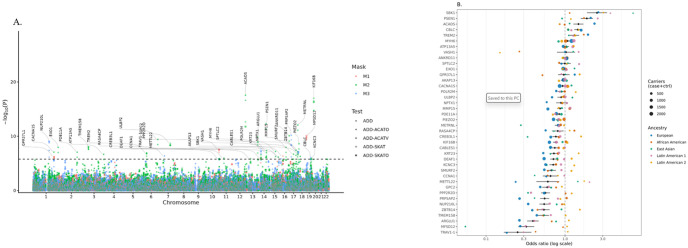
(A) Manhattan plot of gene-based rare-variant association results from burden, SKAT/SKAT-O, and ACAT-V/ACAT-O tests across three VEP-based masks. (B) Ancestry-specific carrier ORs are shown as colored points for the five major ancestry groups. Diamonds denote the pooled OR across all samples, with gray horizontal bars indicating the pooled 95% CI.

Forty genes surpassed the Bonferroni-corrected gene-wide significance threshold (*P* < 1.65 × 10^−6^; 0.05 / 30,276 genes; Figure 1A; **Table S3**). Two of the 40 genes, TREM2^11–14^ and PSEN1^15–18^, are established AD genes with known rare-variant associations, and seven genes lie within 1 Mb of eleven previously reported AD GWAS loci^19–21^, including *TREM2*, *GPC2* (near *NYAP1* and *ZCWPW1*), *POLR2M* (near *ADAM10*), *ANKRD11* (near *PRDM7*), *PRPSAP2* (near *MYO15A*), *CBLC* (near *APOE*), and *KCNC3* (near *CD33* and *SIGLEC11*). We also notice that *ABCA7* showed subthreshold association (*P* = 6.35 × 10^−4^ for M2 and *P* = 1.28 × 10^−4^ for M1), consistent with prior evidence at this locus^22,23^.

Of the 40 genes, eight reached significance in the burden test, which provides a directionally interpretable summary of gene-level effects. *TREM2*, *GPR37L1*, and *SBK1* showed a higher rare-variant burden in AD cases than in controls. In contrast, five genes (*NUP210L*, *PPP2R2D*, *ARGLU1*, *PRPSAP2*, and *MFSD12*) exhibited a significant excess of rare variants in controls compared to cases, suggesting a potential protective role.

To evaluate cross-ancestry consistency, we summarized ancestry-specific rare-variant carrier enrichment across the five major ancestry groups (European, African American, East Asian, Latin American 1, and Latin American 2) using carrier-based odds ratios (ORs). Several genes showed consistent directions across groups; for example, *SBK1*, *CBLC*, and *TREM2* showed risk-increasing directions (OR > 1), whereas *GPC2*, *CCNA1*, *CABLES1*, *METRNL*, *PIEZO2*, and *PDE11A* showed risk-decreasing directions (OR < 1) across major ancestry groups (Figures 1B and **S1**). In contrast, some control-enriched signals in pooled analyses exhibited directionally heterogeneous patterns. For example, *PPP2R2D* showed a protective direction in European, East Asian, and Latin American 1 participants (OR < 1), but showed little evidence of protection in African American participants (OR ~ 1) and trended in the opposite direction in Latin American 2 participants (OR > 1). Overall, genes showing concordant directions of effect across ancestry groups are likely to represent more robust associations. However, directionally heterogeneous patterns can arise when signals are driven by ultra-rare, ancestry-enriched variants, which can differ in frequency and composition across populations and yield unstable direction estimates in smaller strata.

#### AD-Related Trait Analysis

**Figure 2:**
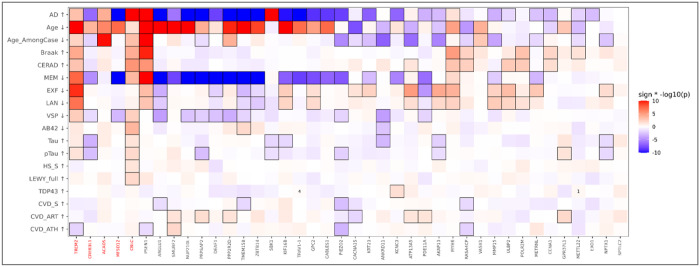
Gene–trait associations from rare-variant carrier analyses. For each gene–trait pair, carriers were compared with non-carriers using Wilcoxon rank-sum tests for quantitative or ordinal traits and Fisher’s exact tests for binary traits. Heatmap shading shows signed −log10(*P*) (red, more adverse trait values in carriers; blue, more favorable trait values in carriers). Cells are outlined for *P* < 0.05; the number of carriers is shown when fewer than five carriers are present. Age_AmongCase denotes the age comparison restricted to AD cases. Arrows indicate the direction of worsening for each trait (down arrow, lower values are worse; up arrow, higher values are worse).

We next examined associations with AD-related traits across the 40 significant genes. Figure 2 summarizes carrier versus non-carrier comparisons for each gene-trait pair (**Table S4**; **Figure S2**), using Wilcoxon rank-sum tests with Hodges-Lehmann location shifts for quantitative or ordinal traits (Age, Age_AmongCase, Braak, CERAD, MEM, EXF, LAN, VSP, Aβ_42_, total tau, pTau_181_, LEWY_full, CVD_ART and CVD_ATH) and Fisher’s exact tests for binary traits (AD, HS_S, TDP43, and CVD_S). Heatmap direction was standardized such that red indicates a shift toward more adverse trait values in carriers and blue indicates a shift toward more favorable trait values; trait labels denote whether worsening corresponds to lower values (Age, Age_AmongCase, MEM, EXF, LAN, VSP and Aβ_42_) or higher values (all other traits shown). *TREM2*, *CREB3L1*, *ACADS*, *MFSD12* and *CBLC* are highlighted as genes replicated in both UK Biobank and ADGC.

Several genes showed broadly adverse trait profiles in carriers, with consistent shifts across multiple domains. In particular, *PSEN1*, *TREM2*, *CBLC* and *MYH6* showed patterns spanning neuropathologic burden (for example, Braak and CERAD), vascular co-pathology measures, and cognitive domains, with additional differences in biomarker readouts for subsets of traits. These cross-domain patterns are consistent with pleiotropic effects of rare variation that track with overall disease burden rather than a single trait.

In contrast, a subset of genes showed more favorable profiles for one or more trait classes. For example, *PIEZO2*, *PDE11A*, *ANKRD11*, and *CREB3L1* showed patterns in which carrier status was associated with shifts toward less adverse trait values. Such “protective” patterns should be interpreted cautiously, as they may reflect resilience biology, trait-specific mechanisms or differences in ascertainment and cohort composition. Nonetheless, these directionally favorable signals motivate follow-up analyses to assess replication and robustness under covariate-adjusted models.

#### Replication in Independent Datasets

Of the 40 significant genes, 15 showed nominal replication in the UK Biobank (UKB; n = 73,413), and 11 showed nominal replication in the Alzheimer’s Disease Genetics Consortium (ADGC; n = 22,846) at *P* < 0.05 (**Table S5**). Five genes replicated in both datasets: *TREM2*, *CREB3L1*, *ACADS*, *MFSD12*, and *CBLC*. When additionally requiring replication under the same annotation mask, two genes (*TREM2* and *ACADS*) replicated in both datasets, with three additional genes replicating in UKB (*NUP210L*, *PIEZO2*, and *PSEN1*) and three additional genes replicating in ADGC (*MFSD12*, *SMURF2*, and *AKAP13*) (Table 1).

In ADSP R5, ACADS carrier status was associated with increased AD risk (Figure 2; OR = 1.33, 95% CI: 1.16–1.53; Chi-square test; *P* = 5.93 × 10^−5^), with a concordant effect in UKB (OR = 1.38, 95% CI: 1.04–1.84; Chi-square test; *P* = 3.19 × 10^−2^). Similarly, TREM2 carriers were more likely to be AD cases in ADSP R5 (Figure 2; OR = 1.26, 95% CI: 1.11–1.43; Chi-square test; *P* = 5.19 × 10^−4^), with a larger effect estimate in UKB (OR = 1.73, 95% CI: 1.53–1.97; Chi-square test; *P* = 8.68 × 10^−18^).

*CBLC* replicated in both datasets but under different masks (ADSP: M3; UKB: M1; ADGC: M2) and lies within 1 Mb of *APOE*; thus, the observed association could be confounded by *APOE* ε4 carrier status. After adjustment for *APOE* ε4, the *CBLC* association markedly attenuated, with the burden test no longer nominally significant (*P* = 0.076), suggesting that the original signal was largely driven by *APOE* ε4.

### Ancestry Concentration and Coalescence Analyses of Rare Variants

#### Ancestry Concentration Analysis

A total of 9,396 variants were observed across the 40 genes. Restricting to variants with at least two carriers (3,448 variants; **Table S6**), 53.83% were ancestry-enriched (defined as ≥75% of carriers from a single ancestry), indicating that a substantial fraction of rare variation is strongly ancestry-concentrated. We next asked whether variants enriched in AD cases were more likely to be confined to a single ancestry. We defined AD-enriched variants as those with ≥2 AD carriers and either no control carriers or an AD/control carrier ratio ≥1, which exceeds the overall case-control ratio in ADSP R5 (AD/control = 12,078/26,308 = 0.459). Among these AD-enriched variants, 349 (63.80%) were “single-ancestry-like” (≥75% of carriers from the same ancestry).

**Figure 3:**
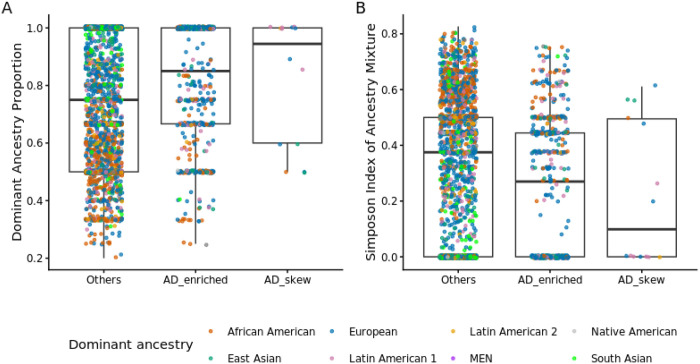
Ancestry concentration of AD-enriched variants in the 40 genes. (A) Dominant-ancestry proportion per variant (maximum carrier fraction in one ancestry) for AD-enriched, AD-skew, and other variants. (B) Simpson diversity index of ancestry mixture across carriers per variant (lower values indicate greater ancestry concentration).

Consistent with this enrichment, AD-enriched variants showed a higher dominant-ancestry proportion than other variants (median 0.86 versus 0.75; Wilcoxon rank-sum test, *P* = 1.85 × 10^−7^), indicating that AD-enriched variants are more strongly ancestry-dominant overall (Figure 3A). Concordantly, the Simpson diversity index indicated lower ancestry mixture among AD-enriched variants than among other variants (Figure 3B).

When stratifying AD-enriched variants by ancestry, we observed that a substantial subset had Latin American 1 as the dominant ancestry. To illustrate this pattern, we examined variants with strong AD skew (≥5 AD carriers and either no control carriers or an AD/control carrier ratio ≥5). We identified 14 such AD-skew variants. Five were confined to Latin American 1: rs1190514189 in *NPTX1*; rs143757018 (p.A399G) and rs116342919 (p.C438T) in *SPTLC2*; rs938226811 (p.L145A) in *VASH1*; and rs63750082 (p.G206A) in *PSEN1*. The remaining variants included rs63749824 (p.A79V), rs63750590 (p.H163A), and rs63750900 (p.A269H) in *PSEN1*, rs145789527 (p.A557C) in *EXO1*, and rs149152296 (p.T39I) in *ANKRD11* in Europeans; rs956022243 (p.I1098V) in *PIEZO2* in African Americans; rs63750083 (p.A431V) in *PSEN1* in Latin American 2; and rs1384004544 (p.G1918L) in *PIEZO2* and rs1322896033 in *ACADS* in East Asians. *PSEN1* contributed the most AD-skew variants. These observations suggest that a subset of AD-enriched rare variants is ancestry-concentrated and may contribute disproportionately to AD burden within specific populations, underscoring the value of diverse ancestry sampling for rare-variant discovery.

#### Coalescence Analysis

To investigate whether AD risk (higher allele frequency in cases) and protective (higher allele frequency in controls) alleles differ in their evolutionary histories, we performed a coalescent-based allele age analysis of GWAS-identified AD variants. We first compiled 97 variants (**Table S7**) from three large AD GWAS^19–21^. For variants not found in our dataset, we selected proxy variants with the highest LD (R^2^) using the LDProxy tool^24^. One variant (rs3822030) lacking a suitable proxy and one indel (rs149080927) filtered by RELATE^25^ were excluded from downstream analyses. In total, this yielded 50 risk alleles and 45 protective alleles, for which we estimated allele ages using RELATE.

**Figure 4:**
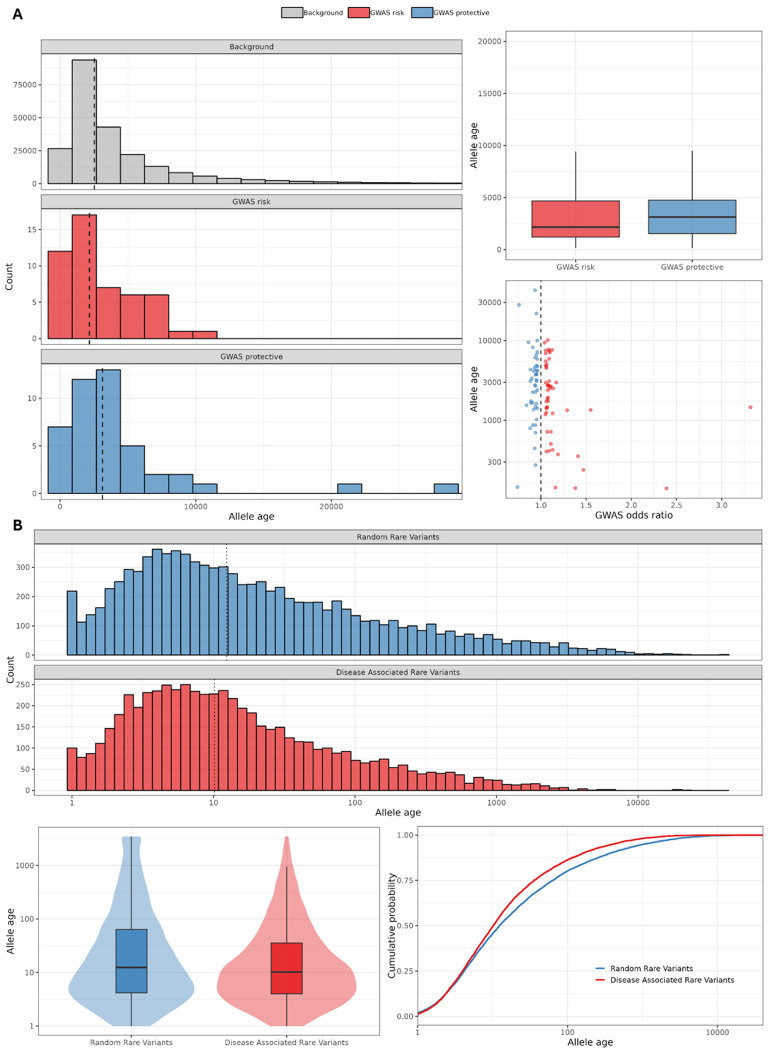
(A) Coalescent-inferred ages of AD GWAS risk and protective alleles versus background variants. Histograms (left) and boxplots stratified by allele-frequency bins (right) show that risk alleles are generally younger than protective alleles. (B) Coalescent-inferred ages of AD-associated rare variants versus rare variants from matched random regions under three annotation masks(M1/M2/M3). Log-scaled histograms (top), violin/boxplots (bottom left), and ECDF curves (bottom right) summarize age distributions; dashed lines indicate medians.

GWAS risk alleles were generally younger than GWAS protective alleles (Figure 4A; Wilcoxon rank-sum test, P = 4.79 × 10^−2^), with median inferred ages of approximately 2,160 and 3,121 generations, respectively. As a reference, we constructed a background set of variants with allele frequency > 0.01 that were in low LD with GWAS variants (R^2^ < 0.1). Compared with this background, risk alleles were significantly younger (*P* = 2.79 × 10^−2^). Background variants had a median age of approximately 2,521 generations, intermediate between risk and protective alleles. Although protective alleles tended to be older than background variants, this difference was not statistically significant (*P* = 2.88 × 10^−1^). Notably, GWAS variants with larger effect sizes (OR > 1.25) were substantially younger, with inferred ages ranging from 140 to 1,458 generations (Figure 4A).

Across the 40 genes prioritized from rare-variant aggregation analyses, we identified 8,101 rare variants, after removing indels and multiallelic variants, of which 6,507 were retained in the RELATE output. After applying quality filters (age CI ratio ≤ 10 and estimated age ≤ 500,000 generations), 5,853 variants remained for downstream analyses. In parallel, we selected 9,247 rare variants with M1/M2/M3 annotations from 40 randomly sampled genomic regions as a matched background set. The median inferred age for variants in the 40 genes was 12 generations, compared with 10 generations for the random regions (Figure 4B; *P* = 3.99 × 10^−14^).

Gene-level analyses highlighted substantial heterogeneity. *PSEN1* (102 variants; median age 6 generations) showed significantly younger inferred ages than the matched background set (**Figure S3**; *P* = 3.63 × 10^−6^), consistent with founder effects. For example, *PSEN1* rs63750082 (p.G206A) has been reported predominantly in families from Puerto Rico and the Dominican Republic, consistent with recent shared ancestry. In contrast, *TREM2* (67 variants; median age 15 generations) did not differ significantly from the random background (**Figure S3**; *P* = 0.67).

### Imputation Reference Panels for Disease Rare Variants

We constructed an ADSP imputation reference panel from the full variant callset using a workflow^26^ with SHAPEIT5^27^ for phasing and Minimac4^28^ for imputation. The resulting panel was designed to maximize coverage of rare coding and noncoding variants observed across ADSP ancestries and to facilitate downstream analyses in cohorts with array genotypes.

**Figure 5:**
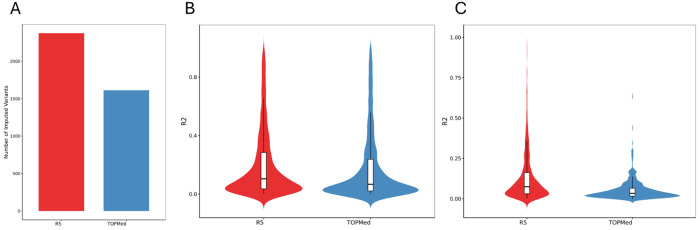
Imputation performance comparison between the ADSP R5 reference panel and TOPMed r2 in the imputed ADGC cohort. (A) Number of imputed rare variants among the prioritized variants in the significant genes. (B) Distribution of imputation quality (R^2^) for variants imputed by both panels. (C) Distribution of imputation quality (R^2^) for variants imputed by only one panel (ADSP R5–only or TOPMed r2-only).

We evaluated the panel by imputing an independent array-genotyped cohort (n = 22,846) from ADGC and compared results with the TOPMed r2 panel. Focusing on rare variants prioritized by our rare-variant aggregation analysis (9,396 variants across 40 genes), our panel imputed 2,374 variants, whereas TOPMed r2 imputed 1,612 variants (Figure 5A). For variants imputed by both panels (1,215 variants), imputation quality measured by R^2^ was higher with our panel (median R^2^ = 0.2352; interquartile range 0.0993 to 0.4891) than with TOPMed r2 (median R^2^ = 0.1578; interquartile range 0.0610 to 0.3905) (Figure 5B). Additionally, 1,159 and 397 were uniquely imputed by our panel and TOPMed r2, respectively. Again, for these uniquely imputed variants, imputation quality measured by R^2^ was higher with our panel (median R^2^ = 0.0748; interquartile range 0.0315 to 0.1622) than with TOPMed r2 (median R^2^ = 0.0338; interquartile range 0.0168 to 0.0659) (Figure 5C). Improvements were most pronounced at the rarest allele frequencies and in ancestries well represented within ADSP.

## Discussion

We analyzed whole-genome sequencing from ADSP Release 5 to evaluate the contribution of rare coding and splicing variation to Alzheimer’s disease (AD) and multiple AD-related quantitative and neuropathologic traits. Using complementary gene-based aggregation tests across functional masks, we identified 40 genes exceeding a Bonferroni-corrected gene-wide threshold, including established AD genes (*TREM2*, *PSEN1*). Several signals showed consistent direction of carrier enrichment across major ancestry groups, and 21 genes showed nominal support in UK Biobank and/or ADGC, with eight genes supported under identical masks. Beyond AD diagnosis, carrier-based analyses across harmonized cognitive, biomarker, and neuropathologic traits indicated that rare-variant burden in a subset of genes tracks with multi-domain disease manifestations. These analyses illustrate the utility of a large, ancestrally diverse WGS resource for rare-variant gene discovery in AD.

A prominent feature of the significant genes was ancestry concentration of rare variants. More than half of the variants observed in these genes were ancestry-enriched, and AD-enriched variants were disproportionately “single-ancestry-like,” consistent with recent origin and restricted geographic or demographic distribution of many rare alleles. These patterns underscore the value of multi-ancestry sequencing for inclusive discovery and caution against over-interpreting ancestry-stratified heterogeneity in carrier odds ratios, which can arise when signals are driven by ultra-rare, ancestry-enriched alleles that differ in frequency and composition across populations. These observations motivate replication strategies that are ancestry-aware and that harmonize qualifying-variant definitions across studies.

Replication in UK Biobank and ADGC further highlights practical constraints in validating rare-variant gene tests across cohorts. Only a subset of signals replicated under identical masks, consistent with limited carrier counts, differences in phenotype definitions and differences in the ascertainment of rare alleles from sequencing versus imputed array data. *TREM2* and *ACADS* showed consistent direction and same-mask replication across cohorts. In contrast, *CBLC*, located near *APOE*, illustrates that gene-based signals can be confounded by nearby common-variant effects, consistent with attenuation after adjustment for *APOE* ε4. Similarly, the absence of replication for *PSEN1* in UK Biobank and ADGC is consistent with extreme rarity, founder effects and ascertainment differences across study designs.

Trait association patterns provide additional context for interpreting gene-level signals. Several genes showed broadly unfavorable carrier profiles across neuropathology, cognition, and vascular co-pathology measures, consistent with pleiotropic effects that align with overall disease burden. Conversely, some genes showed directionally favorable shifts for subsets of traits. These patterns should be interpreted cautiously, given sparse carrier counts and potential cohort and ascertainment differences, and they motivate follow-up using covariate-adjusted models and replication in additional deeply phenotyped datasets.

To place these findings in an evolutionary context, we estimated allele ages using coalescent-based methods. Among GWAS-identified variants, risk alleles were inferred to be younger than protective alleles and younger than a frequency- and LD-matched background set, consistent with enrichment of AD risk among more recent alleles, particularly at larger effect sizes. Extending age estimation to rare variants in the prioritized genes identified modest shifts relative to matched random regions and substantial heterogeneity across genes, consistent with gene-specific demographic histories and founder effects (for example, younger inferred ages for PSEN1 relative to matched background). These results should be interpreted as descriptive summaries conditional on model assumptions, proxy selection, and filtering criteria, but they provide a quantitative framework for relating AD-associated alleles to demographic history across ancestries.

We also constructed an ADSP-based imputation reference panel and evaluated its performance in an independent ADGC array-genotyped cohort. Relative to TOPMed r2, the ADSP-based panel increased the number of prioritized variants that could be imputed and improved imputation quality, with gains most evident at lower allele frequencies and in ancestries well represented in ADSP. These results support a practical route for extending rare-variant discovery to cohorts without sequencing, while emphasizing that imputation remains limited for ultra-rare alleles and is sensitive to reference panel ancestry match.

This study has limitations. Gene-based results depend on annotation masks, allele-frequency thresholds and test choice, which can shift power across genetic architectures and complicate cross-study harmonization. Replication in imputed array data is constrained by reference panel coverage and imputation accuracy, particularly for ultra-rare variants and in underrepresented ancestries. Carrier-based trait comparisons are sensitive to small numbers, multiple testing burden, and cohort-specific trait distributions. In addition, case–control ratios were unbalanced overall and varied across ancestry strata and trait subsets; under sparse carrier counts, imbalance can reduce power and yield unstable ancestry-stratified carrier ORs, motivating cautious interpretation of stratified summaries and replication in well-matched cohorts. Finally, gene-based association implicates loci and variant sets but does not establish causal variants or mechanisms.

In summary, ADSP Release 5 provides a large, multi-ancestry WGS resource for rare-variant association testing in AD. By integrating gene-based analyses across AD diagnosis and AD-related traits, ancestry concentration metrics, allele-age estimates and an imputation panel optimized for ADSP variation, this work expands the set of rare-variant signals relevant to AD and provides tools for replication and downstream studies.

## Methods

### 58,507 Whole-Genome Sequencing Samples

ADSP R5 adds 22,158 new samples from 17 cohorts, bringing the dataset to 58,507 whole genomes across 57 cohorts (**Table S1**). Sample relatedness was assessed with KING^29^. After excluding individuals related at the second degree or closer, n=50,489 remained. Genetic ancestry was inferred with GrafAnc^6^, and each sample was assigned to one of the following groups: African (n=926), African American (n=5,538), East Asian (n= 2,620), South Asian (n=2,660), European (n=26,042), Latin American 1 (such as Puerto Rican in the 1000 Genomes Project; n=7,102), Latin American 2 (such as Colombians and Mexicans in the 1000 Genomes Project, n= 4,407), Middle Eastern and North African (MEN; n=101), Native American (n= 861), or multi-ancestry (n= 232). European ancestry was the largest group (51.6%), followed by Latin American1 (14.1%) and African American (11%) **Table S8**; **Figure S4**).

### Callset with 449 Million Genetic Variants

The raw callset comprised 509,854,643 autosomal variants, and 449,265,450 variants remained after quality control. Each sample harbored an average of 3,612,500 SNVs (95% CI: 3,098,605–4,126,394) and 468,145 indels (95% CI: 382,495–553,794; **Figure S5A**). Across the callset, 51.39% of variants are singletons (53.47% of bi-allelic and 44.40% of multi-allelic variants). In aggregate, 96.58%, 97.42%, and 98.54% of variants have allele frequency (AF) smaller than 0.005, 0.01, and 0.05, respectively (**Figure S5D**). The mean per-sample singleton count is 4,208.49. By ancestry (**Figure S5A**), the highest per-sample singleton means are observed in Middle Eastern and North African (mean = 11,968.70) and South Asian (mean = 9,264.11) samples. Latin American1 (e.g., Puerto Rican and Dominican cohorts) shows the lowest mean (mean = 1,838.44), followed by European ancestry (mean = 3,614.22).

### Annotation

Functional annotation was performed using Ensembl VEP^30^, which assigns each variant one or more Sequence Ontology (SO) consequence terms and an associated impact tier. Because alternate alleles at multi-allelic sites can have distinct effects, we decomposed multi-allelic sites into separate bi-allelic records prior to annotation to retain allele-specific consequences. For downstream analyses, we retained the most severe SO term per variant. If a variant was assigned more than one SOs, for analysis we select the most sever one for it. The selection order is transcript_ablation, splice_acceptor_variant, splice_donor_variant, stop_gained, frameshift_variant, stop_lost, start_lost, transcript_amplification, inframe_insertion, inframe_deletion, missense_variant, protein_altering_variant, splice_region_variant, splice_donor_5th_base_variant, splice_donor_region_variant, splice_polypyrimidine_tract_variant, incomplete_terminal_codon_variant, stop_retained_variant, synonymous_variant, start_retained_variant, coding_sequence_variant, mature_miRNA_variant, 5_prime_UTR_variant, 3_prime_UTR_variant, non_coding_transcript_exon_variant, intron_variant, NMD_transcript_variant, non_coding_transcript_variant, upstream_gene_variant, downstream_gene_variant, TFBS_ablation, TFBS_amplification, TF_binding_site_variant, regulatory_region_ablation, regulatory_region_amplification, feature_elongation, regulatory_region_variant, feature_truncation, and intergenic_variant.

### Rare Aggregation Test

Rare-variant association was performed with REGENIE. We applied complementary gene-based tests (burden, SKAT, and SKAT-O) and, where applicable, Cauchy combination tests (ACAT-V and ACAT-O). Models included covariates for age, sex, sequencing platform, PCR status, and the first 20 genetic principal components (PC1-PC20). Analyses were restricted to variants with minor allele frequency (MAF) < 0.01. For each gene, we constructed three annotation-defined masks: M1 (transcript_ablation, stop_gained, frameshift_variant, stop_lost, start_lost, transcript_amplification), M2 (inframe_insertion, inframe_deletion, missense_variant, protein_altering_variant), and M3 (splice_acceptor_variant, splice_donor_variant, splice_donor_5th_base_variant, splice_region_variant, splice_donor_region_variant, splice_polypyrimidine_tract_variant). Per-gene aggregated tests were then conducted for each mask.

### Ancestry-specific carrier odds ratios and pooled confidence intervals

To visualize cross-ancestry consistency of gene-based rare-variant signals, we generated ancestry-specific carrier odds ratios (ORs) and a pooled estimate per gene using carrier counts. For each gene and variant mask, within each ancestry group we defined *carriers* as individuals harboring at least one qualifying variant in the gene and *non-carriers* as individuals without any qualifying variant. Using the 2×2 contingency table of carrier status (carrier vs non-carrier) by phenotype (AD case vs control), we calculated the ancestry-specific OR. We computed 95% confidence intervals (CIs) using a Wald approximation on the log scale. When any cell count was zero, we applied the Haldane-Anscombe continuity correction by adding 0.5 to all four cells before calculating the OR and CI to avoid infinite estimates.

### Replication Datasets

We evaluated imputation quality and replicated rare-variant aggregation tests using an independent array-genotyped dataset from the Alzheimer’s Disease Genetics Consortium (ADGC; n = 22,846 across 40 cohorts; 10,163 cases and 12,683 controls). Array-genotyped data were imputed using Minimac4 against the reference panel we built in this project. We also performed replication in an independent UK Biobank WGS dataset (UKB; n=73,413; 4,777 cases and 68,636 controls). Controls were required to have an age at recruitment > 65 and no record of any form of dementia. Cases were identified based on health outcomes, hospital diagnoses, self-reported illnesses, and cause-of-death records within UK Biobank.

### ADGC Imputation

Each of 40 ADGC cohorts was first phased using SHAPEIT4 v 4.2.2 and then imputed on the TOPMed r2 imputation server with the TOPMed r2 panel. Concurrently, each phased dataset was also imputed with Minimac4 v4.1.6 using the ADSP-r5 panel. All imputed cohorts were merged into a single dataset by IMMerge. A total of 22,846 samples with recorded AD status (10,163 cases and 12,683 controls) from the merged dataset were used in subsequent analysis.

To compare the imputation quality between the TOPMed r2 and ADSP r5 panels, we calculated harmonized imputation quality scores (R^2^) by combining R^2^ values across all cohorts using Fisher z-transformation for the prioritized imputed rare variants. To compare the panel’s coverage for disease-related variants, we counted the number of imputed disease-related variants in the dataset. Variants with AC > 0 in the dataset were defined as imputed.

### Reference Panel Construction

We first split multi-allelic variants into bi-allelic variants and removed duplicated variants. We then phased 516,026,355 variants derived from the 50,489 whole genome sequenced samples using the SHAPEIT5 v 5.1.1 workflow. Following the workflow, we split each chromosome into several appropriate chunks using Glimpse2 v2.0.0. Common variants (MAF > 0.001) in each chunk were phased first, and all phased chunks from the same chromosome were then ligated together by SHAPEIT5 to form a scaffold. This scaffold was subsequently used to assist in phasing the remaining rare variants (MAF <= 0.001) in each chunk. We then used bcftools v1.19 to concatenate all rare variant phased chunks of each chromosome to generate the final phased dataset. Finally, the phased variant files in VCF format were converted into msav format using Minimac4 v4.1.6 to produce a ready-to-use reference panel.

## Supplementary Material

Supplementary Files

This is a list of supplementary files associated with this preprint. Click to download.

- supptables.pdf
- SuppTextFigures.docx

## Figures and Tables

**Table 1.** Eight genes met the nominal P < 0.05 criterion in UKB and/or ADGC under the same annotation mask used in ADSP R5.

	ADSP R5 (n=38,386)			UKB (n=73,413)			ADGC (n=22,846)		
Gene	Mask (# Variants)	Test	P	Mask (# Variants)	Test	P	Mask (# Variants)	Test	P
ACADS	M2 (154)	ACATV	2.74E-18	M2 (67)	Burden	1.02E-02	M1 (3)	ACATO	2.30E-02
		ACATO	2.46E-17					ACATV	2.30E-02
		SKAT	2.27E-13					SKAT	2.30E-02
		SKATO	1.70E-12					SKATO	2.30E-02
								Burden	2.30E-02
							M2 (47)	SKAT	4.66E-02
TREM2	M2 (77)	ACATO	1.19E-07	M2 (76)	SKAT	7.05E-11	M2 (29)	SKATO	6.50E-09
		SKATO	1.44E-07		ACATO	1.11E-10		ACATO	6.77E-09
		Burden	1.62E-07		SKATO	2.05E-10		SKAT	1.18E-08
		SKAT	4.40E-07		Burden	8.16E-10		ACATV	3.12E-08
					ACATV	1.77E-09		Burden	6.49E-08
NUP210L	M3 (65)	SKATO	9.15E-10	M3 (75)	Burden	3.51E-02			
		SKAT	1.10E-09						
		ACATO	1.58E-09						
		ACATV	4.21E-08						
		Burden	6.86E-07						
PIEZO2	M2 (745)	ACATV	2.44E-09	M2 (776)	SKAT	4.45E-03			
		ACATO	2.19E-08		ACATV	8.22E-03			
					SKATO	8.40E-03			
					ACATO	8.85E-03			
PSEN1	M2 (131)	ACATV	4.96E-13	M2 (94)	SKAT	1.03E-02			
		ACATO	4.47E-12		ACATO	1.28E-02			
		SKATO	7.38E-07		SKATO	1.59E-02			
		SKAT	7.70E-07		ACATV	2.57E-02			
					Burden	3.15E-02			
MFSD12	M1 (28)	SKATO	8.75E-11	M2 (288)	Burden	3.03E-02	M1 (8)	ACATV	3.68E-02
		ACATV	3.07E-10						
		Burden	3.97E-10						
		ACATO	1.46E-10						
		SKAT	1.65E-10						
SMURF2	M2 (118)	ACATV	3.78E-08				M2 (19)	ACATO	1.55E-02
		ACATO	3.36E-07					ACATV	4.87E-02
								SKAT	2.44E-02
								SKATO	1.33E-02
								Burden	3.03E-02
AKAP13	M2 (904)	ACATV	6.92E-07				M2 (276)	Burden	8.53E-03
